# Development, Diversity, and Neurogenic Capacity of Enteric Glia

**DOI:** 10.3389/fcell.2021.775102

**Published:** 2022-01-17

**Authors:** Werend Boesmans, Amelia Nash, Kinga R. Tasnády, Wendy Yang, Lincon A. Stamp, Marlene M. Hao

**Affiliations:** ^1^ Biomedical Research Institute (BIOMED), Hasselt University, Hasselt, Belgium; ^2^ Department of Pathology, GROW-School for Oncology and Developmental Biology, Maastricht University Medical Centre, Maastricht, Netherlands; ^3^ Department of Anatomy and Physiology, The University of Melbourne, Melbourne, VIC, Australia; ^4^ Graduate Institute of Clinical Medicine, College of Medicine, National Taiwan University, Taiwan, Taiwan

**Keywords:** enteric nervous system, glial cells, neurogenesis, gliogenesis, neural crest

## Abstract

Enteric glia are a fascinating population of cells. Initially identified in the gut wall as the “support” cells of the enteric nervous system, studies over the past 20 years have unveiled a vast array of functions carried out by enteric glia. They mediate enteric nervous system signalling and play a vital role in the local regulation of gut functions. Enteric glial cells interact with other gastrointestinal cell types such as those of the epithelium and immune system to preserve homeostasis, and are perceptive to luminal content. Their functional versatility and phenotypic heterogeneity are mirrored by an extensive level of plasticity, illustrated by their reactivity in conditions associated with enteric nervous system dysfunction and disease. As one of the hallmarks of their plasticity and extending their operative relationship with enteric neurons, enteric glia also display neurogenic potential. In this review, we focus on the development of enteric glial cells, and the mechanisms behind their heterogeneity in the adult gut. In addition, we discuss what is currently known about the role of enteric glia as neural precursors in the enteric nervous system.

## Introduction

**FIGURE 1:**
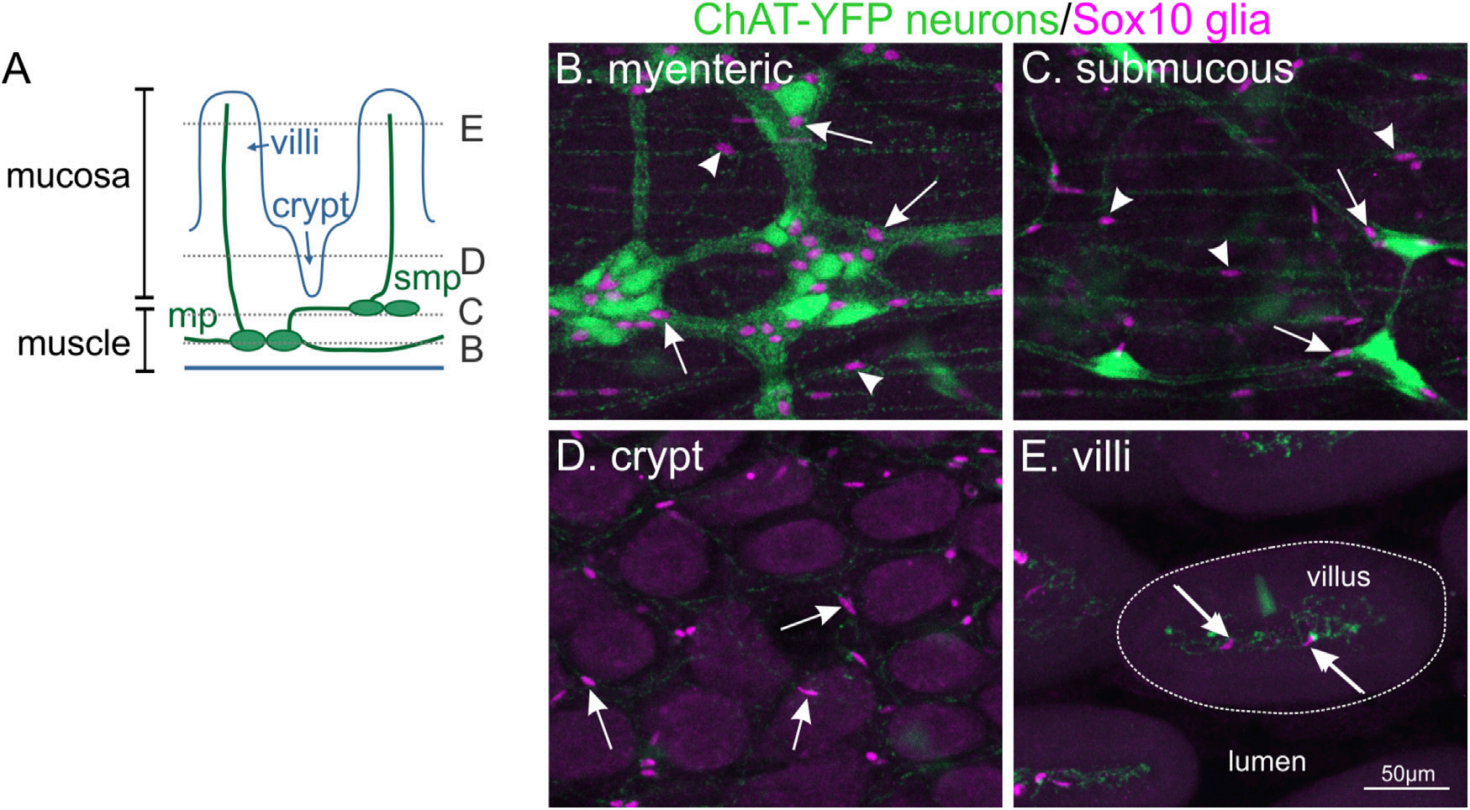
Enteric glial cells are located throughout all different concentric layers of the gut. **(A)**: Diagram of the different cell layers in the small intestine. **(B–E)**: Whole-mount immunohistochemistry on the small intestine of *ChAT-YFP* mouse, where excitatory enteric neurons are labelled with GFP. Ce3D clearing was performed to visualise all tissue layers. The location of Sox10-immunoreactive enteric glial nuclei (magenta, arrows) can be observed with neuronal cell bodies in the myenteric plexus (mp, B), submucous plexus (smp, C) and also associated with neuronal fibres in the muscle layers (arrowheads). In addition, enteric glia are found throughout the mucosa surrounding the crypts (D) and in villi (E).

The enteric nervous system (ENS) is a network of neurons and glial cells located within the wall of the gut that is crucial for control of gastrointestinal function. In mammals, enteric neurons in the stomach, small and large intestines are generally organised in two networks of interconnected ganglia, forming two concentric layers beneath the mucosa (submucous plexus) and between the muscle layers (myenteric plexus; Figure 1). In humans and larger mammals, a tertiary plexus is also present within the mucosa (Furness, 2006). Different types of enteric neurons are wired into dedicated circuits that, together with enteric glia, are responsible for steering smooth muscle activity, mucosal blood flow and secretory activities important for digestive function. Enteric glial cells are found accompanying enteric neurons throughout the gut. They are closely associated with enteric neuronal cell bodies within submucosal and myenteric ganglia in the plexus layers, and are also present in the muscle layers and mucosa, where they generally accompany neuronal processes (Figure 1).

As they are part of the peripheral nervous system and derive from the neural crest, intestinal glia were originally classified as Schwann cells or satellite cells (Cook and Burnstock, 1976). However, in 1980, a landmark study by Jessen and Mirsky showed that these glial cells express glial fibrillary acidic protein (GFAP), whose expression at the time was only found in astrocytes of the central nervous system (CNS) (Jessen and Mirsky, 1980). Considering them as a separate type of peripheral glia, Gabella was the first to define them as “enteric glial cells”. A number of common markers for enteric glia have since been identified, including the transcription factors Sox10 (Young et al., 2003) and Sox2 (Heanue and Pachnis, 2011; Belkind-Gerson et al., 2017), the calcium binding protein S100B (Ferri et al., 1982), and more recently the myelin associated protein, proteolipid protein 1 (Plp1) (Rao et al., 2015).

**FIGURE 2:**
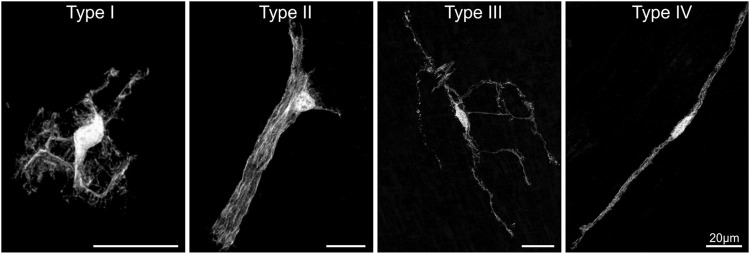
The morphological enteric glial cell subtypes. Individual enteric glial cells with GFP labelling throughout their cell body to visualise their morphology. Sparse labelling of individual cells was achieved using the *Sox10-cre;MADM* transgenic mouse system. Adapted from (Boesmans et al., 2015).

There are multiple types of glial cells in the enteric nervous system. A detailed description of local heterogeneity of enteric glia can be found in recent reviews (Rosenberg and Rao, 2021; Seguella and Gulbransen, 2021). Briefly, the current classification scheme for enteric glia, initiated in 1994 by Hanani and Reichenbach, is based on the morphology of guinea pig myenteric glia and can be linked to locations within the gut wall (Hanani and Reichenbach, 1994). Type I enteric glia are protoplasmic star-shaped cells within myenteric (Type I_MP_) and submucosal (Type I_SMP_) ganglia (Figure 2). Type II enteric glia are “fibrous” cells with long processes running along neuronal processes in interganglionic fibre tracts (Hanani and Reichenbach, 1994). These morphologies have been confirmed in several studies since then and were examined in further detail using genetic approaches (Boesmans et al., 2015; Rao et al., 2015). Type III glia are multipolar, with most cells having 4 major processes. They are present in both the lamina propria (Type III_mucosa_) and outside the primary component of the plexus layers which consist of ganglia and interganglionic connectives (Type III_MP/SMP_) (Savidge et al., 2007; Gulbransen et al., 2012; Boesmans et al., 2015). And lastly, Type IV enteric glia are bipolar cells associated with neuronal processes in the circular and longitudinal smooth muscle layers (Vanderwinden et al., 2003; Gulbransen et al., 2012; Boesmans et al., 2015). Since this classification into four enteric glia subtypes mostly stems from studies on the murine ileum and colon, it will be important to investigate how it holds for other gut regions and whether similar enteric glia subtypes are present in other species, including human. Confirming the current classification, a recent study reported the presence of similar morphological subtypes in the murine oesophagus (Kapitza et al., 2021). Furthermore, both mucosal, intramuscular and myenteric enteric glia have been described in the human colon (Liu et al., 2013; Graham et al., 2020).

Data from single cell or single nuclei RNA-seq studies have also classified enteric glia into different groups based on their transcriptional profiles. Zeisel et al., identified 7 different subtypes of enteric glia in juvenile mice (P21-23), including 1 cluster that was proliferative (Zeisel et al., 2018). Drohklansky et al., have described 3 different subtypes of enteric glia in adult mice, and 6 in the adult human ENS, 3 of which were patient-specific cell clusters (Drokhlyansky et al., 2020). Varying expression of the P2Y12 receptor was highlighted, which could correspond to the differences in responses to the neurotransmitter ATP by different enteric glia subtypes (Boesmans et al., 2015; Drokhlyansky et al., 2020). And in two studies published earlier this year, a thorough interrogation of gene expression patterns in the foetal human gastrointestinal tract was conducted, including examination of enteric glia (Elmentaite et al., 2021; Fawkner-Corbett et al., 2021). However, further research is required to investigate if the transcriptionally distinct enteric glia subtypes can be linked with the morphological classification scheme.

Our current understanding on the role of enteric glia in ENS activity and gastrointestinal function overall has been carefully described in several recent reviews (Neunlist et al., 2014; Coelho-Aguiar et al., 2015; Grubišić and Gulbransen, 2017; Rosenberg and Rao, 2021; Seguella and Gulbransen, 2021). Because enteric glial cells are present throughout the different layers and regions of the gut, facing very different local microenvironments, it is likely that they are also functionally specialized in order to execute locally-adapted tasks. While this might be reflected by their differential morphology or marker gene expression, so far, the majority of studies investigating enteric glial function has not considered specific subtypes. However, based on the emerging transcriptomic data and the identification of subtype-specific promotor and/or enhancer activity, this might be possible in the near future. In this context, it will be crucial to determine the extent of plasticity in both space and time of a morphological and functional subtype collection present within a specific niche at a given moment.

The biology of enteric glia holds many unanswered questions and several of these pertain to understanding the contribution of phenotypic plasticity to functional heterogeneity during both homeostasis and ENS dysfunction (Holland et al., 2021). Trying to help answer these questions, this review assesses the developmental origins of enteric glia and the appearance of different subtypes of enteric glial cells. In addition, we discuss the current understanding of their role as neural stem cells of the gut.

## Enteric Glial Development: Neural Crest Origins

Like neurons of the ENS, enteric glial cells arise from neural crest derived cells that migrate into the gut during development. The majority of the ENS comes from neural crest cells at the “vagal” level of the neural tube (adjacent to somites 1–7) (Yntema and Hammond, 1954; Le Douarin and Teillet, 1973). However, the vagal neural crest does not comprise a single, homogenous population. Cells from different levels along the anterior-posterior axis have marked differences in their abilities to generate the ENS, and contribute to other cell types (Hutchins et al., 2018; Simkin et al., 2019). Using chick embryos, precise ablation and transplantation experiments have shown that neural crest cells adjacent to somites 1–2 and 6–7 have limited contribution to the ENS (Burns et al., 2000); whilst those adjacent to somite 3 have the ability to form a complete ENS, even in the absence of all other vagal crest cells (Barlow et al., 2008). Similarly, in the mouse, neural crest cells from somites 3–7 form the majority of the ENS, with limited contribution by somite 1–2 neural crest cells, which were identified mostly in the oesophagus and stomach (Espinosa-Medina et al., 2017). Sacral neural crest cells also contribute to ENS of the colon (Le Douarin and Teillet, 1973; Burns et al., 2000) but their migration takes place later than that of the vagal crest. Sacral neural crest cells also leave the neural tube at E9.5, but cells accumulate and “pause” at the pelvic ganglia before entering the developing colon from the ventrolateral side at E13.5, migrating along nerve fibres from the pelvic ganglia (Wang et al., 2011).

Schwann cell precursors are a neural crest-derived stem cell pool found on the peripheral nerves that further contribute to the same cell types as the neural crest itself, including melanocytes, sympathetic and parasympathetic neurons, neuroendocrine cells, (Petersen and Adameyko, 2017), and ENS (Uesaka et al., 2015; Uesaka et al., 2021). Schwann cell precursors from both cranial and trunk regions migrate into the gut along the vagal and sympathetic nerves, respectively (Uesaka et al., 2015; Espinosa-Medina et al., 2017). Arriving in the gut after its initial colonisation by vagal neural crest cells (NCCs), trunk Schwann cell precursors make up approx. 20% of the enteric neurons in the colon (Uesaka et al., 2015; Uesaka et al., 2021). In the small intestine they are not present in the myenteric plexus, but contribute to a small proportion (5%) of submucous neurons. Interestingly, Schwann cell precursors make a greater contribution to enteric neuron numbers when there is reduced density of the ENS, such as in Hirschsprung disease (described below (Uesaka et al., 2021)).

Combined, neural crest cells arriving in the gut that give rise to the ENS are commonly referred to as “enteric neural crest-derived cells” (ENCCs). Almost as soon as they arrive in the gut, ENCCs start to differentiate into neurons and glia (Baetge and Gershon, 1989; Young et al., 2003). It is assumed that the neural crest from all these different levels of the neural tube have a comparable capacity to form both enteric neurons and glia, however, the generation of enteric glia has never been specifically examined separately from that of enteric neurons. Whether one source of neural crest cells has a higher neurogenic vs gliogenic potential, and therefore makes a larger contribution to neurons vs glia in the ENS, is currently not known. How Schwann cell precursors contribute to the constellation of enteric glial cells in homeostatic conditions is also unclear. However, given their transcriptomic similarities and extensive plasticity (Dulac and Le Douarin, 1991; Rao et al., 2015), distinguishing between intestinal glia that arise from different sources is turning out to be a challenge, and might even be irrelevant from the perspective of ENS function.

The ENS is one of many cell types that reside in the gastrointestinal tract. The gut consists of concentric layers of different cells originating from the endoderm, which gives rise to the epithelial cells lining the gut, and the mesoderm, which produce the majority of the mesenchymal derivatives in the gastrointestinal tract, such as the smooth muscle cells, and stromal cells (Guiu and Jensen, 2015). Endodermal cells are also the source of enteroendocrine cells that are embedded in the epithelial layer (Andrew et al., 1982), which respond to various chemical and mechanical stimuli from the gut lumen and communicate to enteric neurons and glia. Interestingly, endoderm lineage cells have also been observed to contribute to the ENS (Brokhman et al., 2019). Using *Pdx1-cre* and *Sox17-cre* mice to drive reporter gene expression, derivatives of the endoderm lineage were observed in the myenteric plexus, and found to express neuronal markers. Interestingly, no glial markers (GFAP or S100B) were expressed by these endoderm-derived ENS cells (Brokhman et al., 2019). Although the majority of mesenchymal cell derivatives in the gut are thought to arise from the embryonic mesoderm, a subset of vagal neural crest cells has been identified to express mesenchymal genes in both chick and mouse (Zeisel et al., 2018; Ling and Sauka-Spengler, 2019). This may not be as surprising as neural crest cells give rise to many different cell types, including bone, melanocytes, and mesenchymal cells such as cardiac muscle cells (Jiang et al., 2000; Soldatov et al., 2019); however, this had not previously been observed within the gut. In the mouse, at least, the contribution of the vagal neural crest to the mesenchymal cell pool appears to be very small (Morarach et al., 2021). However, further investigation of these different developmental directions can provide important information on the gene regulatory pathways governing ENS differentiation.

## Evolution of Enteric Glia and Investigation of Other Model Organisms

The ENS is often referred to as the “second brain” as it functions independently from the central nervous system to control many aspects of gastrointestinal function. However, in terms of evolution, the ENS appears to be the “first brain”, as its structure of interconnecting neurons resembles what we deduce a primitive nervous system looks like, and which can be found in many simple invertebrates, such as hydra and jellyfish (Furness and Stebbing, 2018; Gilbert, 2019). In hydra, one of the most basic living animals that consists of two main body cell layers surrounding a central gut-like structure, the nervous system is made up of a “nerve net”, consisting of two layers of neurons dispersed amongst the epithelium (Gründer and Assmann, 2015). However, there is no evidence of glial cells in hydra or other *Cnidarians* (Gründer and Assmann, 2015; Verkhratsky et al., 2019). The most primitive animals with glial cells are flatworms, which belong to the phylum *Xenacoelomorpha* (Biserova, 2000; Verkhratsky et al., 2019).

Looking at another branch of evolution, the ENS of insects is quite distinct from that of vertebrates, particularly as they do not come from a neural crest origin. Although there are clearly differences between the multitude of various insect species, there are several common themes, which have been described in detail previously (Copenhaver, 2007). The ENS is primarily present in the foregut, with fewer ganglia in the midgut, while the hindgut receives innervation from the CNS. In some species, such as *Drosophila melanogaster*, the midgut mostly lacks enteric neurons (Miguel-Aliaga et al., 2018). Unlike vertebrates, the insect ENS precursors arise from the ectoderm of the foregut itself. Neuronal precursors generated from the epithelium then migrate to populate the ganglionated regions of the gastrointestinal tract. This has been well-documented in the *Manduca sexta* moth (Copenhaver and Taghert, 1990, 1991), and also *Drosophila melanogaster* (Hartenstein et al., 1994). During *Manduca* development, enteric glial cells also arise from the neurogenic epithelium of the foregut following neurogenesis. They are produced as a last wave of precursor cells which migrate in close association with the previously generated neurons, which then proliferate and differentiate to form glial cells that wrap around the enteric neurons and nerve fibres (Copenhaver, 1993). In *Drosophila*, much of the “enteric nervous system” is part of the stomatogastric nervous system, which also innervates the pharynx and other muscles (Miguel-Aliaga et al., 2018). Like in *Manduca*, neurons of the enteric/stomatogastric system arise from the epithelium of the foregut, which migrate and differentiate into various neuron subtypes and glia (Hartenstein et al., 1994; Forjanic et al., 1997). Stomatogastric glial cells also come from the same epithelium, and interestingly, they migrate and guide the projections of axons (Forjanic et al., 1997). While not much research has focused on the genetic mechanisms driving stomatogastric gliogenesis specifically, several of the pathways identified in CNS neurogenesis and gliogenesis could be conserved across the different regions (Crews, 2019). Thus far, the *pnt* gene, which is important for many other aspects of *Drosophila* development, has been shown to control stomatogastric gliogenesis (Forjanic et al., 1997). Investigation in this field could be highly fruitful as there are many common genetic mechanisms used by both *Drosophila* and vertebrates, despite the differences in origin of the ENS progenitors. For example, the receptor tyrosine kinase Ret and the proneural transcription factors in the achaete-scute complex (AS-C) are important for both *Drosophila* and vertebrate ENS development (*Ascl1* is the vertebrate homologue of achaete-scute) (Hartenstein et al., 1996; Myers et al., 2018). The ease of genetic manipulation of *Drosophila* and the availability of many transgenic models make it a highly attractive model organism to investigate enteric gliogenesis (Forjanic et al., 1997; Hernández et al., 2015).

A key model vertebrate organism in investigation of the ENS is zebrafish. There are several key structural differences between the ENS of zebrafish compared to that of mammals. The zebrafish ENS does not contain separate plexus layers and enteric neurons are present as single cells rather than in ganglia (Wallace et al., 2005; Shepherd and Eisen, 2011; Heanue et al., 2016; Ganz, 2018; Kuil et al., 2020). Recently, there has been some debate regarding the presence and developmental origin of enteric glia in zebrafish. Earlier studies have reported the presence of GFAP in the zebrafish gut but whether the labelled structures represent ENS components is not clear (Hagstrom and Olsson, 2010; Baker et al., 2019). In 2020, based on the notion that all neural-crest derived cells in the gut were found to express neuronal markers by 5 days post fertilisation (dpf), El-Nachef and Bronner suggested that there are no “classical” enteric glia in zebrafish (El-Nachef and Bronner, 2020). No S100B-immunoreactive cells were observed, and although GFAP-immunoreactivity appeared to be present, there were no labelled cell bodies. In addition, using *in situ* hybridisation, they did not detect *plp1* in the ENS, and surprisingly did not observe *sox10* expression either, despite previous indications that *sox10* is expressed at both the transcript and protein level in the ENS at these stages (Taylor et al., 2016; Kuwata et al., 2019). Instead, El-Nachef and Bronner suggest that trunk neural crest cells, which migrate later into the gut, give rise to neurons after embryonic development and also in cases of gut injury (El-Nachef and Bronner, 2020). Identified as Schwann cell precursors, these are proposed to be the neural stem cell population of the zebrafish ENS (see below).

Interestingly, McCallum et al. demonstrate that there is a population of enteric glia in adult zebrafish, consisting of all 4 morphological subtypes of enteric glia seen in mammals (Boesmans et al., 2015; McCallum et al., 2020). They show that approximately 15% of cells in the gut at 7dpf are non-neuronal, increasing to 35% in the adult zebrafish. As observed in the study by El-Nachef and Bronner, these cells do not express the canonical enteric glia markers S100B and GFAP, but were nonetheless classified as enteric glia based on their transcriptomic profile, morphology and location. Apart from alternative experimental approaches, the differences in timing of the two studies could prove to be crucial to explain the apparently conflicting observations. One possibility is that the enteric glial cells identified by McCallum et al. do not come from the vagal neural crest, but rather migrate into the gut as Schwann cell precursors after 5dpf, as identified by El-Nachef and Bronner. However, this seems unlikely as McCallum et al. describe *her4:GFP* as a marker of adult enteric glial cells, and also show *her4:GFP* expressing cells emerging from the vagal migratory stream (McCallum et al., 2020). Further studies will likely clarify the developmental origins of these enteric glia in zebrafish.

In addition to zebrafish, the more basal jawless vertebrate, lamprey, has emerged as an important tool in investigation of ENS development and neural crest evolution. A recent study in sea lamprey (*Petromyzon marinus*), has shown that the ENS arises not from the vagal neural crest, but instead, the trunk neural crest cells (Green et al., 2017). This provides an interesting narrative for ENS development through vertebrate evolution. Schwann cell precursors contributing to the mammalian ENS may be a primitive state retained from early vertebrates, where trunk neural crest form the majority of the ENS (Green et al., 2017). In addition to cell migration, analysis of gene regulatory networks guiding neural crest specification in these different model organisms has led to important insights on developmental processes during ENS formation (Nikitina and Bronner-Fraser, 2009; Martik and Bronner, 2021). Investigation of different animals in our evolutionary tree will further our understanding of how enteric glia develop and function.

## Factors That Control Glial Differentiation

Following entry into the gut, there is extensive ENCC proliferation and differentiation, as well as further migration to colonise the entire gastrointestinal tract. In the mouse, the timeline of ENCC migration and neuronal differentiation has been well-established (Young and Newgreen, 2001; Obermayr et al., 2013; Hao et al., 2016; Nagy and Goldstein, 2017; Rao and Gershon, 2018). The appearance of glial markers has also been described, however, there is still limited knowledge on how bipotent ENCCs make the critical decision to become either neurons or glia. A mix of cell-intrinsic and environmental factors play important roles in guiding ENS development, including gliogenesis (Charrier and Pilon, 2017; Pawolski and Schmidt, 2020). Many of these pathways have been described in detail elsewhere in the context of overall ENS development and defects in these pathways are implicated in Hirschsprung disease, a neurodevelopmental disorder that arises from the failure of neural crest cells to colonise the entire gastrointestinal tract (described in detail below) (Heanue and Pachnis, 2007; Amiel et al., 2008; Sasselli et al., 2012; McKeown et al., 2013; Liu and Ngan, 2014; Avetisyan et al., 2015; Nagy and Goldstein, 2017). Here we specifically focus on their roles in enteric glial development and differentiation.

Future research in this area is likely to benefit from the increasing amount of data describing gene expression patterns in both mature and developing enteric glia (Lasrado et al., 2017; Zeisel et al., 2018; Drokhlyansky et al., 2020). Single cell RNA-seq performed on ENS cells at E12.5 identified 3 main populations of cells: a “neuronal” cluster, a “undifferentiated/gliogenic” cluster, and a small, third cluster which expressed a low level of all genes examined (Lasrado et al., 2017). Further analysis of genes in the undifferentiated/gliogenic cluster, and comparison to the mature ENS data sets, will help identify factors that are involved in enteric glia differentiation and maintenance.

### Sox10, a key transcription factor in neural crest and enteric glia development.

**FIGURE 3:**
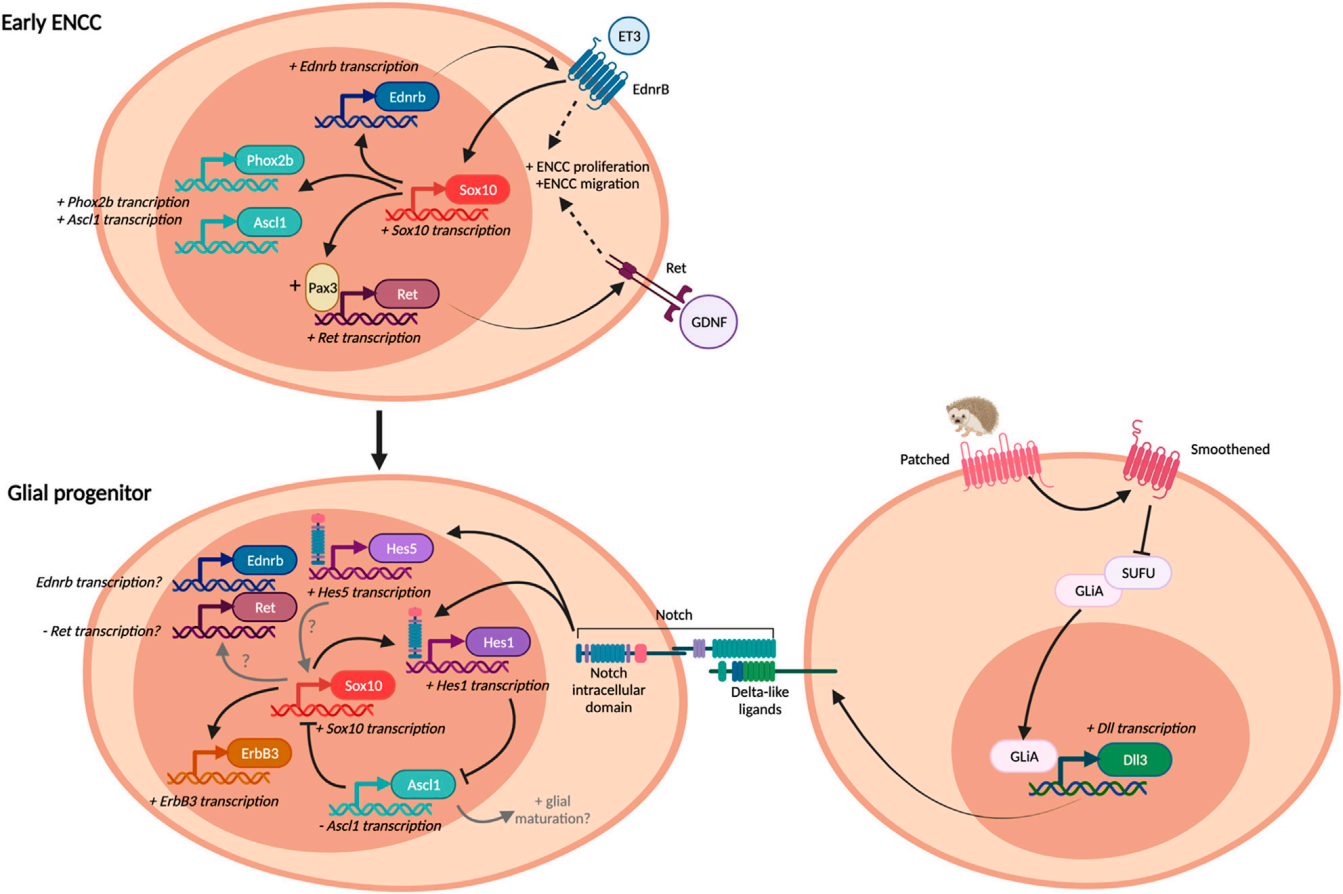
Pathways in gliogenesis. Some pathways are commonly employed both in early ENCC development and enteric glial differentiation, but may play different roles in each situation, depending on the presence of other factors. Adapted and expanded from (Liu and Ngan, 2014).

*Sox10* plays crucial roles in both neural crest specification and the differentiation and maintenance of peripheral glial cells (Britsch et al., 2001; Paratore et al., 2001). *Sox10* is an important neural crest gene, and is expressed by undifferentiated ENS progenitors, even prior to their emergence from the neural tube (Kuhlbrodt et al., 1998; Southard-Smith et al., 1998). It is crucial for ENS development, as mice that lack *Sox10* expression have total intestinal aganglionosis (Southard-Smith et al., 1998). As a transcription factor, it controls and modulates the expression of several key genes for early ENS development, including *Ret* (Lang et al., 2000; Lang and Epstein, 2003) and *Ednrb* (Zhu et al., 2004). Intriguingly, it also promotes the expression of several transcription factors important for neuronal differentiation, such as *Phox2b* and *Ascl1* (Kim et al., 2003). The role of Sox10 at early stages of ENS development appears to differ from its later task in directing enteric glial development. During early embryogenesis Sox10 appears to be responsible for promoting ENCC proliferation and migration, as well as the induction of a wide array of both neuronal and glial genes (Figure 3).

As ENCCs differentiate, enteric neurons downregulate *Sox10* expression, but Sox10 is maintained in the majority of enteric glia (Young et al., 2003). Among other genes, Sox10 induces expression of the neuregulin receptor, *ErbB3* in neural crest cells. ErbB3 signalling is important for promoting enteric glial proliferation and differentiation, as *ErbB3* knockout mice fail to develop enteric glia at E12.5 (Riethmacher et al., 1997). ErbB3 is activated by its ligand, glial growth factor 2 (GGF2), which is synthesised by the gut mesenchyme. *ErbB3* expression is also promoted by bone morphogenetic protein (BMP) signalling (Chalazonitis et al., 2011). Interestingly, mice that lack *ErbB3* expression in their neural crest also have decreased neuronal density along the entire length of the gut during embryonic development, however, this can be attributed more to decreased migration rather than changes to glial differentiation (Espinosa-Medina et al., 2017). How Sox10 maintains its dual roles both in promoting glial differentiation and maintaining multipotency remains unknown. The timing and level of *Sox10* expression are likely to be important (Kim et al., 2003). Oscillating expression of various pro-neuronal and pro-glial transcription factors has been observed in multipotent progenitors, with the expression of individual transcription factors being sustained as they make a fate choice (Imayoshi et al., 2013). This model is likely to also take place in differentiating ENCCs, and *Sox10* expression levels could provide a means to discriminate between enteric glial cells with or without neurogenic potential.

In addition to *Sox10*, several other Sox genes are also expressed by ENCCs and are important for ENS development. *Sox8*, *Sox9* and *Sox10* belong to the *SoxE* family of transcription factors, which are highly conserved in vertebrates and generally show overlapping expression (Reiprich and Wegner, 2015), including in the ENS (Maka et al., 2005). However, there are still clear differences in the function of these different transcription factors. For example, in mice, loss of function of *Sox10* cannot be replaced by *Sox8* expression. Attempted rescue of *Sox10* deletion using a knock-in *Sox8* model restored the ENS in the oesophagus and stomach, but the ENS failed to develop in the small and large intestines (Kellerer et al., 2006). How enteric glia were affected in the rescued regions was not examined, however, Schwann cells and other peripheral glial cells appeared to be restored (Kellerer et al., 2006). While *Sox2* belongs to the *SoxB1* group, its expression closely overlaps with that of *Sox10* in the ENS (Heanue and Pachnis, 2011), and is also often used as a marker to detect enteric glia in the adult ENS (Jonscher and Belkind-Gerson, 2019; Morarach et al., 2021). From an evolutionary perspective, how the different *Sox* genes can compensate for each other has been examined in a recent comprehensive study, where different *Sox* genes were expressed in *sox10-knockout* zebrafish embryos, including *SoxE* genes from the jawless vertebrate, lamprey, and the invertebrate, lancelet (Lee et al., 2016). The lamprey *Sox9* orthologue (*PmSoxE3*) and the single *SoxE* gene identified in the invertebrate lancelets (*Branchiostoma floridae*) were able to induce ENS neuron formation (Lee et al., 2016), indicating they have similar function to the vertebrate *Sox10*.

### Notch and Hedgehog Signalling Pathways

Notch and hedgehog are also important regulators of both ENS development and gliogenesis (Liu and Ngan, 2014). It’s well known that Notch signalling plays an important role in cell fate determination through development (Heitzler and Simpson, 1991; Ho et al., 2020). Notch is a transmembrane receptor that is activated by its ligand, Delta, which is also a transmembrane protein. Therefore, cell-cell contact is required for this juxtacrine signalling (Figure 3). In mammals, there are 4 notch receptors (Notch 1–4), and 5 ligands (delta-like1, 3, 4, and jagged 1, 2). In the ENS, *Notch1*, *Delta-like 1* (*Dll1*) and *Dll3* are expressed, and some cells also express *Notch4*, *Dll4* and *Jagged1* (Okamura and Saga, 2008). Activation of Notch1 by its ligand, Dll1 or Dll3, results in transcription of target genes, including the *Hes* (hairy and enhancer of split) family of transcriptional repressors (Bray, 2016). *Hes1* is expressed in the developing ENS, as well as in non-ENCCs in the gut at E10.5 (Okamura and Saga, 2008) and has been shown to repress the expression of pro-neural genes, such as *Ascl1* (aka *Mash1*) (Chen et al., 1997). Notch activation was found to promote *Sox10* expression, through indirect action via *Hes1* and its repression of *Ascl1* expression (Okamura and Saga, 2008), as Ascl1 represses *Sox10* expression (Kim et al., 2003). During ENS development, *Ascl1* expression is found both in Sox10+ progenitors and enteric neurons. *Ascl1* has been shown to be important for neuronal differentiation in the oesophagus (Sang et al., 1999), stomach and intestines (Blaugrund et al., 1996), particularly for some subtypes of enteric neurons (Memic et al., 2016). While *Ascl1* is generally considered to be a pro-neural gene, it also contributes to enteric glial differentiation. In *Ascl1* knockout mice, the proportion of S100B + enteric glia is decreased in the colon and distal small intestine, but more proximal gut regions were not affected (Memic et al., 2016). This indicates that there are likely to be further actions of *Ascl1* on enteric glial differentiation, in addition to repression of *Sox10*.

In recent transcriptomic studies, *Hes1* expression appears to dominate in the progenitor pool of the embryonic ENS, while enteric glia express *Hes5*, as well as the closely related transcription factors *Hey1* and *Hey2* (Morarach et al., 2021). Interestingly, *Hes6* expression was more specific to neuroblasts (Morarach et al., 2021). Expression of these different subsets of transcription factors is likely to be important for the segregation of the neuronal vs glial differentiation lineages, however, how these differences are initiated remains unknown. Elsewhere in the nervous system, different ligands for Notch produce different downstream intracellular signals, which can be responsible for switching on the expression of different transcription factors (Bray, 2016). How this is controlled in the ENS has not yet been investigated. In the central nervous system, *Hes5* expression has been identified in radial glial cells (Ruan et al., 2021) and is also important for maintaining oligodendrocyte precursors as proliferating cells, preventing their differentiation (Sock and Wegner, 2021). Interestingly, Hes5 achieves this by preventing the interaction of Sox10 with its downstream myelinating genes. How these actions of Hes5 relate to enteric glial differentiation remains to be investigated. In zebrafish, the orthologue of *Hes5*, *her4.3* was used as a marker to identify enteric glial cells through development and in adulthood, and as described later, Notch signalling was found to persist in the mature ENS for controlling neurogenesis (McCallum et al., 2020).

Activation of the hedgehog signalling pathway decreases neurogenesis in the gut (Sukegawa et al., 2000; Fu et al., 2004; Reichenbach et al., 2008; Liu and Ngan, 2014), and promotes premature gliogenesis (Ngan et al., 2011). Hedgehog signalling acts through the Patched (Ptch) and Smoothened (Smo) transmembrane receptors to promote the transcription factors Gli proteins (*Gli1, 2, 3*) to activate transcription of their targets (GliA, Figure 3). Gli proteins can also act as transcriptional repressors (GliR), and different levels of hedgehog signalling result in different ratios of Gli activators/Gli repressors (Liu and Ngan, 2014). Activation of the hedgehog signalling pathway has been shown to increase *Hes1* expression. This was identified to be mediated via activation of *Dll1* expression, thereby there is convergence of the hedgehog and notch signalling pathways (Figure 3) (Ngan et al., 2011). Gli activation can also promote *Sox10* expression (Liu et al., 2015), thereby promoting gliogenesis.

### GDNF (Glial Derived Neurotrophic Factor)—Ret

GDNF-Ret signalling plays many crucial roles in ENS development, including ENCC migration and proliferation (Sasselli et al., 2012; Lake and Heuckeroth, 2013). GDNF is produced by the gut mesenchyme, and ENCCs express its receptor, Ret, and co-receptor, GFRa1. In the absence of GDNF, Ret, or GFRa1, there is no ENCC migration beyond the stomach and results in total intestinal aganglionosis (Schuchardt et al., 1994; Pichel et al., 1996; Sanchez et al., 1996; Enomoto et al., 1998). As one of the master regulators of ENS development, GDNF-Ret signalling interacts with many other pathways, including those downstream of Sox10 and Endothelin-3 (described below). Ret signalling and Sox10 act synergistically during early ENS development, as both are important for neural crest migration and proliferation (Heanue and Pachnis, 2007; McKeown et al., 2013; Avetisyan et al., 2015; Nagy and Goldstein, 2017). In addition, Sox10 has been shown to act together with Pax3, another important neural crest transcription factor, to promote *Ret* expression (Lang et al., 2000; Lang and Epstein, 2003).

The role of GDNF-Ret signalling changes through development, and attenuated timing and location of increased GDNF expression has different impacts on the ENS (Wang et al., 2010). *Ret* becomes upregulated in neurons and downregulated in enteric glia (Young et al., 1999). Further, mosaic deletion of *Ret* expression has been shown to increase the expansion of cells lacking *Ret,* i.e., enteric glial cells, at later stages of ENS development (Lasrado et al., 2017). This suggests that *Ret* expression is important to commit bipotential progenitors to the neuronal lineage and that *Ret* downregulation by enteric glia may be important for sustaining their proliferation. How the actions of Sox10 upregulate *Ret* expression during early ENS development fit in with the later downregulation of *Ret* in enteric glia has not been examined. As described above, it’s likely that there are changes in the roles of these transcription factors and signalling pathways as the ENS matures, which could be important for modulating further changes in *Ret* expression and progenitor maintenance.

### Endothelin 3—Endothelin Receptor B

Endothelin-3 signalling, acting via its receptor Endothelin receptor B (EdnrB), inhibits neuronal differentiation in the ENS, thereby maintaining ENCCs in a progenitor state (Hearn et al., 1998) (Figure 3). This allows ENCCs to proliferate and migrate to colonise the entire gastrointestinal tract. In the absence of Endothelin-3 signalling, ENCCs prematurely differentiate into neurons, and fail to migrate to the end of the colon. *Endothelin-3* and *Ednrb* knockout mice lack enteric neurons in the terminal colon and are often used as models of Hirschsprung disease (Baynash et al., 1994; Hosoda et al., 1994). Thus far, how endothelin signalling impacts glial differentiation specifically *in vivo* has not been examined. Endothelin-3 signalling interacts with many pathways, including Ret, Sox10, and retinoic acid (Barlow et al., 2003; Gisser et al., 2013; Watanabe et al., 2017). Endothelin signalling acts to maintain Sox10 levels *in vivo* (Stanchina et al., 2006), and Sox10 promotes *Ednrb* expression in ENCCs migrating into the distal colon (Zhu et al., 2004). In addition, Endothelin-3 signalling enhances Ret signalling to promote the proliferation of ENCCs (Barlow et al., 2003). More recently, *in vitro* cultures have shown that for ENCCs isolated from embryonic mouse and rat gut, endothelin signalling decreases both neuronal and glial differentiation (Gisser et al., 2013; Watanabe et al., 2017). Therefore, it’s possible that endothelin signalling acts on Sox10 only in the context of maintaining the ENCC progenitor population, and other factors are needed to promote enteric glial differentiation. It would be interesting to examine how Endothelin 3—Ednrb signalling impacts on the maintenance of the neural precursor population in the adult gut.

### Foxd3

Foxd3 is also a key transcription factor in neural crest specification. It’s expressed by pre-migratory neural crest cells and deletion of *Foxd3* in neural crest cells results in a complete intestinal aganglionosis (Teng et al., 2008). Like Sox10, Foxd3 is also important for maintaining neural crest cell multipotency and loss of Foxd3 results in cells moving towards a mesenchymal, rather than neural, fate (Mundell and Labosky, 2011). In addition, Foxd3 has a specific role in enteric gliogenesis. Although ubiquitously expressed in early neural crest cells, its expression is downregulated in enteric neurons and maintained in enteric glia (Mundell et al., 2012). Furthermore, deletion of *Foxd3* at later embryonic stages results in reduced enteric glial differentiation (Mundell et al., 2012). How Foxd3 achieves different roles during development is unknown, but likely involves differing interactions with histone deacetylases (HDACs) and other DNA modification proteins for transcriptional repression (Krishnakumar et al., 2016; Respuela et al., 2016). Indeed, epigenetic regulation is an emerging field of study in the understanding of ENS development (described below).

### Nr2f1

*Nr2f1* was recently identified to be an important gene in gliogenesis (Bergeron et al., 2016). Nr2f1 is an orphan nuclear receptor transcription factor, whose role in ENS development was identified in an insertional mutagenesis screen. Mutant *Spot* ^*Tg/Tg*^ mice were identified to have premature glial differentiation. In these mutants, *Nr2f1/2* expression was identified to be over-activated by the inhibition of a non-coding repressing element (Bergeron et al., 2016). Interestingly, *Hes5* expression was also found to be downregulated in *Spot* ^*Tg/Tg*^ mutants (Charrier and Pilon, 2017).

### Epigenetic and miRNA-Mediated Gene Regulation

Differential gene expression is also controlled by the ability of the transcriptional machinery to access the DNA. This includes changes to either the DNA or the proteins involved in DNA folding, that either prevent or allow gene silencing and gene activation. DNA methylation and histone modifiers are the most studied forms of epigenetic regulation (Torroglosa et al., 2016). In addition, non-coding RNAs such as microRNAs are known to influence gene expression post-transcriptionally (Bartel, 2004). Epitranscriptomic mechanisms, where the transcribed RNA is modified to influence its stability and translation (Frye et al., 2018), have yet to be examined in the context of ENS development.

Current knowledge on epigenetic modulation of the ENS function was recently reviewed in relation to both Hirschsprung disease and irritable bowel syndrome (Gazouli et al., 2016; Jaroy et al., 2019). Analysis of the aganglionic gut from Hirschsprung patient resections have allowed comparison of expression levels of genes and proteins involved in epigenetic silencing during ENS development, such as DNA methyltransferases (DNMTs) and histones. Increased methylation, involving addition of methyl groups by DNMTs, prevents access to the DNA, thereby silencing the gene. Specific DNMTs, such as DNMT3b, and methyl binding proteins, such as MeCP2 have been identified to play a role in neural crest and ENS development (Zhou et al., 2013; Villalba-Benito et al., 2017). Knocking down *dnmt1* and *uhrf1* (another DNA binding protein which recruits histone deacetylases) in zebrafish leads to reduced ENCC colonisation of the gut (Ganz et al., 2019). In addition, the methylation of a promoter area has been shown to influence expression of key ENS development genes, such as *Ret* (Munnes et al., 1998) and *Ednrb* (Tang et al., 2013). How changes in DNA methylation and folding combines with the action of specific transcription factors to lead to changes in gene expression patterns during ENS development is an emerging field of study.

MicroRNAs also play an important role in ENS development. These small noncoding RNAs control gene expression post-transcriptionally, by base-pairing to partially complementary sequences in target messenger RNAs (Bartel, 2004). Our knowledge of microRNAs and their role in ENS development is only beginning (Kang et al., 2021), also mostly starting from uncovering their expression and possible roles in Hirschsprung disease (Sergi et al., 2016; Villalba-Benito et al., 2021).

While epigenetics, epitranscriptomics and the role of microRNAs in enteric gliogenesis haven’t yet been investigated specifically, it is likely that such regulatory mechanisms are involved. Recently, chromatin shaping has been identified to be important for predetermining vagal neural crest into their various lineages (Ling and Sauka-Spengler, 2019). Enteric glia were found to derive from progenitors that have both E2 and NC2 enhancer activity, but not EC2 or NC2 alone. How this relates to the control of specific genes remains to be investigated further. Interesting information can also be inferred from studies on Schwann cells, other peripheral glia, and also oligodendrocytes, which all have partially overlapping transcriptomic profiles, including expression of the key transcription factor *Sox10* (Rao et al., 2015). In Schwann cell development, the histone deacetylases 1 and 2 (HDAC1/2) have been found to be important for induction of *Pax3* expression, which maintains high levels of *Sox10* during specification, and subsequent expression of brain fatty acid binding protein (*Fabp7* aka B-FABP) and myelin protein zero (*Mpz*) (Jacob et al., 2014). While enteric glia are non-myelinating they do express myelin-associated genes, some transiently during development, and others also in the adult ENS (Lee et al., 2001; Young et al., 2003; Rao et al., 2015). Therefore, it’s quite possible that similar pathways are involved in enteric glial differentiation. In zebrafish, downregulation of *hdac1* has been shown to delay ENCC migration and reduce enteric neuron numbers (Ignatius et al., 2013), however, the effects on enteric glia have not yet been examined.

## Appearance of Enteric Glial Markers During Development

During mouse ENS development, there is sequential acquisition of the various enteric glial markers. ENCCs enter the gut at E9.5, already expressing Sox10 (Anderson et al., 2006). At E11.5, a subpopulation of ENCCs express the glial progenitor marker brain fatty acid binding protein (B-FABP) (Young et al., 2003). At E14.5, S100B is present (Young et al., 2003) and GFAP at E16.5 (Rothman et al., 1986). Not surprisingly, there is an increase in the proportion of S100B + ENCCs during embryonic development, presumably as more progenitors differentiate into enteric glia (Hao et al., 2017a). *Plp1*, also a marker of adult enteric glia, is expressed by E12.5 (Rao et al., 2015; Lasrado et al., 2017). Interestingly, *Mpz*, another gene involved in myelin formation, is transiently expressed during embryonic development (E12—E14) (Lee et al., 2001), but is not present in adult enteric glia (Jessen and Mirsky, 1983). However, single cell RNA-seq studies show that *Mpz* expression is still present in the Schwann cell precursor pool of the ENS at E18.5 (Morarach et al., 2021). Of note, in the chick gut glial differentiation appears to occur earlier than in the mouse, as GFAP immunoreactivity is present close to the migratory wavefront (Conner et al., 2003).

Although glial cells in the zebrafish gut don’t appear to express the classical enteric glial markers, in previous studies, GFAP-immunoreactive cells were identified in the gut at 3 dpf (Hagstrom and Olsson, 2010). Zebrafish enteric glia were found to exhibit Notch activity, and could be labelled with the transgenic Notch activity reporter *her4.3:EGFP*. Using this reporter, GFP+ glia were identified at 60 h post fertilisation, increasing in expression at 4 dpf (McCallum et al., 2020).

During human embryonic development, p75+ ENCCs are present in the developing foregut at 4 weeks of gestation, and colonisation of the hindgut is complete by week 7 (Wallace and Burns, 2005). S100B + immunoreactive glia and S100B gene expression have been observed in the human gut at week 12, but no studies have examined any earlier ages. Interestingly, GFAP expression was relatively low in the human gut from week 12–16, indicating that perhaps GFAP expression is also delayed compared to S100B (McCann et al., 2019).

In postnatal development, enteric glia were found to decrease in density in ganglia over the weaning period in mice (2–6 weeks) (Parathan et al., 2020), similar to what was found for enteric neurons. This is most likely due to an overall decrease in the density of enteric ganglia as the gut grows and the distance between ganglia spreads. Interestingly, a large proportion of submucosal HuC/D+ neurons, as well as a small subpopulation of myenteric neurons, were found to co-express the glial markers Sox10 and S100B (Parathan et al., 2020). It is likely that these are newly differentiated neurons which are in the process of switching off glial marker expression. As the submucous plexus generally develops later than the myenteric, it would be interesting to examine whether this phenomenon is more prevalent in the myenteric plexus at earlier postnatal ages.

## The Appearance of Enteric Glial Subtypes

**FIGURE 4:**
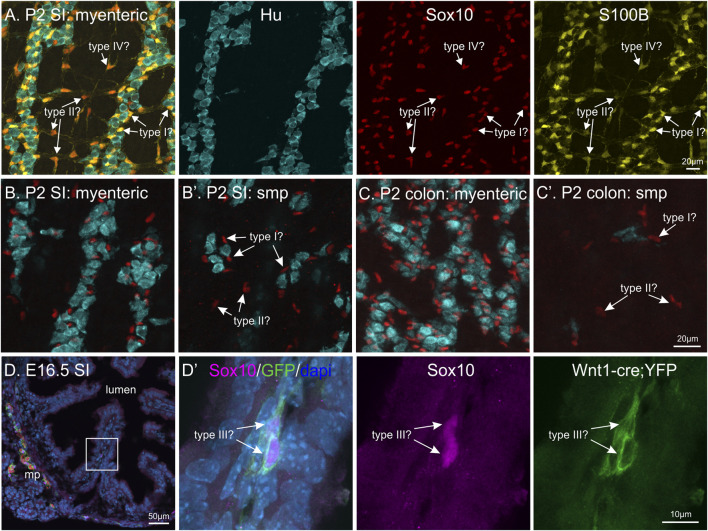
Differentiation of enteric glial subtypes during ENS development? **(A–C)**: Whole-mount postnatal day 2 (P2) murine gut with immunohistochemical staining against HuC/D (blue), Sox10 (red) and S100B (yellow). By P2, enteric glia are present in different locations in the various gut layers: in the myenteric and submucous (smp, arrows) plexus in both the small intestine (SI) and colon, and also surrounding neurons within ganglia. Should these enteric glia be classified as type I, II, III or IV based on their locations? **(D)**: Transverse cryosection of E16.5 small intestine from *Wnt1-cre;R26R-YFP* mice, where all neural crest-derived cells express YFP. Immunohistochemical labelling against Sox10 (magenta), HuC/D (red), YFP (green) and dapi (blue) was performed. Inset on left image is magnified in the following panel. Sox10+ cells can be observed in some villi (arrows), these could be classified as either type III enteric glia or progenitor cells.

Whilst the acquisition of these immunohistochemical markers of enteric glia is now well-described, the development of glial “activity” and the differentiation of the various subtypes of enteric glia has not been investigated. The developmental appearance of enteric glia subtypes, using the currently accepted morphological classification, is currently unknown. During embryonic development, the majority of enteric glia first appear at the level of the plexus layers of the gut. At E14.5 and E16.5, S100B+ enteric glia are closely associated with enteric neurons and progenitors at the myenteric plexus (Hao et al., 2017a). Gangliogenesis, i.e. the separation into individual ganglia, begins at embryonic stages (Young et al., 1998) and, like many events during ENS development, progresses in a rostral-to-caudal wave (Epstein et al., 1991). Therefore, enteric glia could be classified as either Type I, II, III or IV based on their location with respect to the myenteric and submucosal ganglia, or presence within the mucosa (Figure 4). However, how their morphology relates to adult enteric glial cells residing in these locations and how the structural maturation of the plexus layers influences enteric glia phenotype is not known. Mucosal enteric glia (Type III) have been identified from postnatal development and the presence of microbiota is key for attracting enteric glia into the gut mucosa layers (Kabouridis et al., 2015). In the adult ENS, mucosal glia are believed to be constantly replaced by cells migrating from the plexus layers, but how this relates to progenitor/enteric glia proliferation in the plexus layers is not known. Recently, single cell RNA-seq of the developing human gut have shown that there are already multiple subtypes of enteric glia before 12 weeks gestation (Elmentaite et al., 2021; Fawkner-Corbett et al., 2021). There is abundant expression of *Sox10* and *S100B* at weeks 6–11 gestation, with lower levels of *Fabp7* (B-FABP), and very little *Gfap*. Interestingly, *S100B* expression was not restricted to glial cells, but also found in progenitors, neuroblasts and some lineages of enteric neurons (Elmentaite et al., 2021). This has also previously been identified in mouse ENS data sets (Zeisel et al., 2018), however, how these lower expression levels relate to translation and detection of the S100B protein remains to be investigated further.

While the molecular mechanisms underlying enteric glial cell heterogeneity are largely unknown, most findings argue that the array of enteric glia subtypes is determined by microenvironmental cues rather than genetic lineage restrictions. For instance, although enteric glia express a differential set of “common” markers *in vivo*, they quickly upregulate such molecules when isolated and cultured *in vitro* (Boesmans et al., 2015). Also, genetic fate mapping demonstrated that the expression of GFAP by individual enteric glia is extremely dynamic and echoes their high level of phenotypic plasticity (Boesmans et al., 2015). Further proof comes from clonal analysis of ENS morphogenesis, where all 4 morphological enteric glial cell subtypes were present in “glia-only” clones *in vivo* (Lasrado et al., 2017).

When enteric glia in the developing ENS are able to receive input from enteric neurons and when they become “functionally active” has not yet been investigated. In the adult ENS, enteric glia communicate via connexin hemichannels and receive input from enteric neurons through purinergic signalling (Gulbransen et al., 2012; Boesmans et al., 2013; McClain et al., 2014; Fung et al., 2017; Boesmans et al., 2019). One study has shown that between E11.5—E14.5 in the embryonic mouse, calcium waves propagate between both enteric neurons and Sox10 + ENCCs, mediated by purinergic signalling between adjacent cells (Hao et al., 2017b). It’s possible that enteric glia participate in this form of wave communication, however, whether S100B+ cells were involved was not conclusively identified.

## Development of Enteric Glia in the Absence of Enteric Neurons

ENCC progenitors first start to differentiate into neurons before the appearance of enteric glial cells. Whether the presence of enteric neurons influences glial differentiation has not been investigated. Interestingly, enteric glial cells are present in the absence of enteric neurons, for example, in Hirschsprung Disease. As described above, Hirschsprung disease is a developmental disorder that occurs when neural crest cells fail to colonise the entire gastrointestinal tract (Amiel et al., 2008). Without neural crest precursors, the ENS fails to form in the caudal bowel. The severity of the disease depends on the length of the aganglionic region, which, in the majority of cases, occurs in the colon, but can sometimes extend into the small intestine (Kawaguchi et al., 2021). In the absence of an ENS, there is no control of gut motility and faecal contents cannot move past the aganglionic region, accumulating to form a megacolon in affected infants. Hirschsprung disease occurs at an incidence of 1:5,000 live births. Surgical resection of the aganglionic bowel is a necessary and life-saving procedure (De La Torre and Langer, 2010). Studies of resected gut from patient samples, as well as animal models of Hirschsprung disease have shown that in the aganglionic region, segments of innervated bowel can be present (O'Donnell and Puri, 2010). These so called “skip segments” contain enteric neurons and glia, which were thought to arise from sacral neural crest (Burns et al., 2000; O’Donnell and Puri, 2010). More recently, Schwann cell precursors and vagal neural crest cells migrating across the mesentery have been shown to contribute to skip segments (Yu et al., 2020; Uesaka et al., 2021).

**FIGURE 5:**
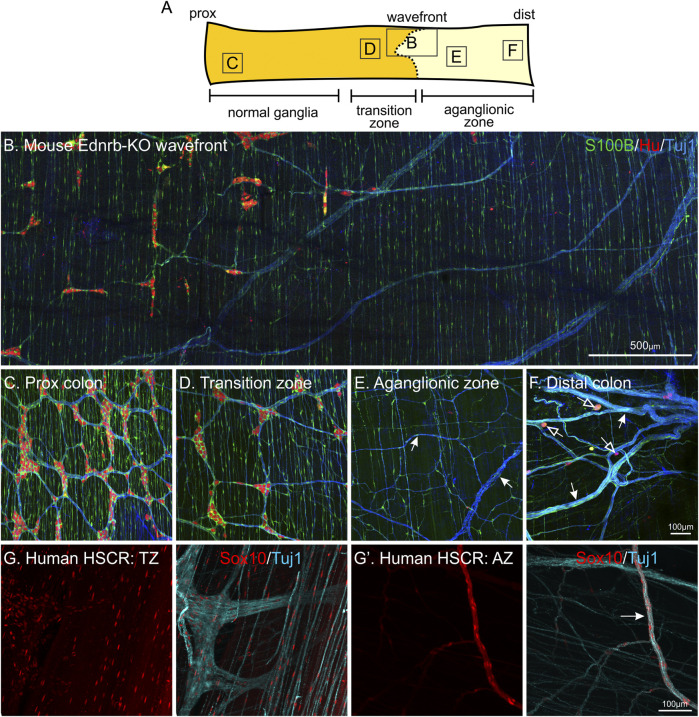
Enteric glia are present in the gut in the absence of enteric neurons: the aganglionic zone of Hirschsprung Disease (HSCR) tissue. Whole-mount immunohistochemistry of *Ednrb-KO* mouse (A–F) and human HSCR patient tissue (H-H′). **(A)**: Diagram of mouse colon including the ENS wavefront and showing the regions with “normal” ganglia, the transition zone (TZ), and the aganglionic zone (AZ). **(B–F)**: Representative images from each of these regions, following immunohistochemistry against S100B (green), HuC/D (red) and neuronal class III β−tubulin (Tuj1, blue). The majority of S100B + glia in the aganglionic region follow nerve bundles, both large and small (arrows). Sometimes, individual Hu + neurons are present in the aganglionic zone (open arrows). NB. These are not “skip segments” as there are no clear ganglia, instead, they are just a handful of individual neuronal cell bodies. **(G-G’)**: Patient HSCR samples labelled against Sox10 (red) and Tuj1 (blue). In the aganglionic zone, Sox10 + glia are also associated with nerve processes. The same proximal—distal orientation has been maintained in all tissues.

In addition to skip segments, glial cells have also been identified in the aganglionic colon where neurons are completely absent (Graham et al., 2020; Soret et al., 2020). This has been demonstrated using both the *Ednrb*-knockout (*Ednrb-KO*) transgenic mouse model of Hirschsprung disease (Lane, 1966; Hosoda et al., 1994; Soret et al., 2020), as well as patient samples taken from aganglionic resected gut (Graham et al., 2020) (Figure 5). The enteric glial cells closely associated with extrinsic peripheral nerves are likely to arise from Schwann cell precursors, which migrate along these nerves throughout the body (Petersen and Adameyko, 2017). However, they appear to have a very limited capacity to differentiate into neurons as very few neuronal cell bodies are observed in the aganglionic zone (Figure 5). One recent study has investigated the potential of driving these enteric glia to innervate the aganglionic gut using GDNF (Soret et al., 2020). The application of GDNF to the aganglionic rectum was able to stimulate these glia to proliferate and generate enteric neurons, thereby restoring gut motility and ensuring survival of a number of different transgenic mouse models of Hirschsprung disease (Soret et al., 2020). This is highly promising as a potential adjunct treatment. Although defects in *RET* account for 50% of familial cases of Hirschsprung disease (Amiel et al., 2008), the migration and differentiation of Schwann cell precursors in the gut does not appear to be hampered by the lack of *Ret* expression (Uesaka et al., 2021). However, why these enteric glial cells in the aganglionic colon fail to differentiate into neurons in the absence of exogenous GDNF, and whether they can be channelled as a form of cell therapy for the treatment of Hirschsprung disease in larger mammals remains to be investigated further.

## Enteric Glia as “Stem Cells” in the Adult ENS

The identity of the neural stem cell population in the adult ENS has been a topic of debate. Neural crest stem cells in the peripheral nervous system were described in 1992 by Stemple and Anderson (Stemple and Anderson, 1992). Cells expressing the low-affinity nerve growth factor receptor, p75 on their surface were isolated using fluorescent activated cell sorting (FACS) and were found to be multipotent in culture (Stemple and Anderson, 1992). These “neural crest stem cells” were also identified and examined in the embryonic gut (Lo and Anderson, 1995; Kruger et al., 2002; Kim et al., 2003), and were found to persist in the adult gastrointestinal tract (Kruger et al., 2002). The neurogenic capabilities of these stem cells were mostly assessed *in vitro*, by their ability to differentiate into neurons and glia in culture. To investigate their neurogenic potential, methods to grow neurospheres were developed, both with and without using FACS to isolate p75+ cells (Schäfer et al., 2003; Almond et al., 2007), using reporter mouse lines where ENCCs are labelled (Hotta et al., 2013), and also from Hirschsprung patient tissue following gut resections (Lindley et al., 2009). Much research has been dedicated to investigating the use of these enteric neural progenitors in cell therapy as a treatment for Hirschsprung disease (Burns et al., 2016).

Whether there are proliferating stem cells in the adult ENS, and the identity of these cells has been the subject of several recent studies. In rodent model studies, enteric glial cells have been suggested to act as the “stem cell” population in the ENS. Enteric glia do proliferate, although at low levels, in the healthy rat gut. Following a 6-weeks daily pulse of BrdU and a 6-weeks chase, 2.8% of S100B+ enteric glia were found to be proliferative (Joseph et al., 2011). Single cell RNA-seq studies indicate that a subpopulation of enteric glia in juvenile (P21-23) mice express markers of proliferative cells (Zeisel et al., 2018). However, as described above, their precise localisation in the gut, as well as how they correlate with the current morphological classification remains to be investigated further.

Enteric glia can also transdifferentiate into neurons without the need for proliferation. Using benzalkonium chloride (BAC), a detergent that denervates small regions of the small intestine, lineage-marked glial cells (displaying Sox10 promotor activity) were found to change to expressing neuronal markers 1 month following denervation (Laranjeira et al., 2011). These cells were thought to migrate into the denervated region from nearby ganglia, but did not arise from proliferating cells (Laranjeira et al., 2011). Similarly, studies investigating enteric neurogenesis in the context of inflammation have found that enteric glial cells transdifferentiate into enteric neurons (Belkind-Gerson et al., 2017). In experimental models of colitis, increased density of enteric neurons was observed, however, in the absence of cellular proliferation (Joseph et al., 2011; Belkind-Gerson et al., 2017). The newly generated enteric neurons were found to differentiate from cells expressing enteric glial markers *Sox2* and *Plp1* (Belkind-Gerson et al., 2015; Belkind-Gerson et al., 2017).

In a controversial study by Kulkarni et al., 80% of enteric neurons was found to be replaced every 2 weeks in adult mice (Kulkarni et al., 2017). Extensive proliferation of ENS cells was identified using incorporation of the thymidine analogues IdU and CldU. Proliferating cells were shown to express *Nestin*, a commonly used marker of stem cells in the CNS. However, whether *Nestin* can be used as a marker of stem cells in the ENS is still unclear. *Nestin* expression has been identified in both enteric neurons and glial cells, both in studies investigating the localisation of the Nestin protein (Eaker and Sallustio, 1994; Vanderwinden et al., 2002; Azan et al., 2011; Cantarero Carmona et al., 2011) as well as using *Nestin-GFP* reporter mice (Belkind-Gerson et al., 2013; Grundmann et al., 2016). While Kulkarni et al. suggest that the proliferating population does not express Sox10, some of these cells were positive for the enteric glia markers S100B and GFAP. Still, whether they represent a dedicated neural stem cell population, or a subset of enteric glia remains to be clarified. More recently, in a follow up study by the same group, toll-like receptor (TLR) activation via the microbiome was found to be important for the differentiation of neurons from Nestin+ cells in the adult ENS (Yarandi et al., 2020). The degree of proliferation proposed by these two studies has not yet been replicated in any other labs, and further investigation is needed to confirm how enteric neurogenesis proceeds in the mature adult gut.

The zebrafish ENS has been suggested to have a higher regeneration capacity compared to mammalian models and neurogenesis has been described in 4 key studies, both during larval development (Kuwata et al., 2019; El-Nachef and Bronner, 2020; McCallum et al., 2020; Ohno et al., 2021) and also in the adult (McCallum et al., 2020). At 3-4dpf, ENCCs have just migrated to the caudal end of the gut (Shepherd et al., 2004). By using an inducible *zebrabow* transgenic line, individual ENCCs could be identified at these stages and their progeny followed using both timelapse imaging and posthoc immunohistochemistry (Kuwata et al., 2019). Dividing cells were observed to give rise to all combinations of neuron/neuron, neuron/Sox10+, and Sox10+/Sox10+ daughter cell dyads, indicating that multipotent progenitors are present.

At the later age of 10–15 dpf, the gut is fully colonised and a mixture of both enteric neurons and Sox10+ cells are present (Ohno et al., 2021). Using an infrared laser-evoked gene operator system to remove the ENS at a specific somite level, colonisation of the ablated region was observed (Ohno et al., 2021). Nearby enteric neurons extended neurite “bridges” into the ablated region, and both neuronal and non-neuronal ENCCs migrated towards and occupied the affected area. Cells colonising the ablated region consisted of newly generated cells that proliferated following laser ablation, as well as newly differentiated neurons. Although the density of cells did not reach its full capacity 10 days following ENS ablation, this data reveals important information about the proliferative and regenerative capacity of ENS cells. Two different models have been proposed to explain the identity of these regenerative cells in the zebrafish ENS.

*De novo* neurogenesis following laser ablation of the ENS in the zebrafish larvae was also observed by El-Nachef and Bronner. This neurogenesis was attributed to Schwann cell precursors that migrated into the gut and promoted by the 5-HT_4_ receptor agonist, prucalopride (El-Nachef and Bronner, 2020). As described above, this study focused on investigating neurogenesis at 5 dpf, immediately following vagal NCC colonisation of the gut. When demonstrating that new neurons derive from non-resident neuronal precursors the authors made use of *phox2b* regulatory elements to drive expression of the Kaede reporter. However, *phox2b* activity has been shown to be restricted to committed neuronal progenitors and enteric neurons (Roy-Carson et al., 2017). Therefore, this raises the possibility that in their experiments, uncommitted resident ENS progenitors that were present at this stage were not labelled, and thus escaped photoconversion. In a similar study, postnatal neurogenesis was identified, but attributed to enteric glia located within the gut (McCallum et al., 2020). Enteric glia in the adult zebrafish gut (3 months old) were found to be proliferative, incorporating the thymidine analogue, EdU, after a 3-days pulse. Only about 10% of enteric glia were found to be cycling, suggesting that the majority of enteric glia remain quiescent, with a small activated population that enter the cell cycle. In addition, a small population of neurons were identified to have taken up EdU during this time, and were found in couplets with non-neuronal cells, suggesting that they arose due to proliferation and subsequent differentiation of glia. Notch activity was found to have an important role in the regulation of both gliogenesis during development, and also glial proliferation and neurogenesis in the mature adult ENS (McCallum et al., 2020). As described above, although these results appear to suggest different cellular sources, it is clear that neurogenesis in the adult zebrafish comes from non-neuronal cells located in the gut, likely representing enteric glia. To what extent Schwann cell precursors contribute to this progenitor population remains to be investigated. Nonetheless, these studies have opened up new avenues for further investigation into the activation pathways involved in adult neurogenesis in both zebrafish and mammalian ENS models.

### Signalling Pathways Responsible for Neurogenesis in the Adult Enteric Nervous System

Several signalling pathways have been implicated in neurogenesis in the adult ENS under physiological and pathological conditions. Serotonin (5-HT) is a prominent signalling molecule in the gut and is produced primarily by mucosal enterochromaffin cells and neurons (Gershon, 2013). Along with critical roles during development, serotonin has been found to be involved in adult neurogenesis. This role appears to rely on signalling through the 5-HT_4_ receptor that is found on both enteric neurons and glia. Neurogenesis was initially described in response to treatment with a 5-HT agonist in the adult gut (Liu et al., 2009). BrdU was detected in a subset of cells expressing enteric glia markers that were located extraganglionically and subsequently migrated into the ganglia where they acquired a neuronal phenotype. When 5-HT_4_ receptor KO animals were treated with the 5-HT agonist, neurogenesis was not observed (Liu et al., 2009). More recently, colitis induced neurogenesis was found to be blocked by 5-HT_4_ receptor antagonism (Belkind-Gerson et al., 2015). Using *Sox2-GFP* reporter mice, which label enteric glia, Belkind-Gerson and colleagues showed that these new neurons arose from enteric glial cells. In a follow up study using the same *Sox2-YFP* reporter mice, they demonstrated that new neurons did not incorporate EdU, suggesting Sox2+ glial cells underwent direct neuronal transdifferentiation (Belkind-Gerson et al., 2017). In a germ-free mouse model, microbiota colonization in the adult stimulated proliferation of Nestin+ cells and maturation to neurons via an increase in 5-HT production and activation of the 5-HT_4_ receptor (De Vadder et al., 2018). In addition, as mentioned above, microbiota have also been implicated in regulating neurogenesis from Nestin+ cells via Toll-like receptor 2 (Yarandi et al., 2020).

In zebrafish models, neurogenesis from glia has been found to involve the Notch signalling pathway. As described above, McCallum and colleagues (2020) found that *her4.3:GFP*, a Notch activity reporter, labelled enteric glial cells in the adult zebrafish. These cells underwent proliferation and occasionally gave rise to neurons. Notch signalling was shown to be involved in this process as treatment with a Notch inhibitor led to the loss of *her4.3* expression. Loss of *her4.3* was associated with increased glial proliferation and enteric neurogenesis, although it was not determined whether Notch signalling had a direct effect on neurogenesis or whether the increase in neurons came indirectly as a result of increased glial proliferation (McCallum et al., 2020).

GDNF, which plays many crucial roles in ENS development, is also implicated in postnatal neurogenesis (Soret et al., 2020). In this study, GDNF was delivered via enema to several mouse models of Hirschprung disease. GDNF treatment was found to stimulate the migration and proliferation of Schwann cells associated with extrinsic nerves which subsequently gave rise to new enteric neurons and glia. The effect of GDNF was dependent on the GDNF receptor, GFRa1, however GDNF signalling in Schwann cells appeared to be mediated by NCAM rather than GDNF’s more well-known signalling partner, Ret. Whilst a subset of new enteric neurons and glia appeared to arise from Schwann cells, a significant percentage of induced neurons did not express the Schwann cell lineage reporter nor did they incorporate EdU (Soret et al., 2020), suggesting that both neuronal transdifferentiation and differentiation from non-enteric precursors can occur in the same system simultaneously. Together, these studies investigating adult neurogenesis in rodents and zebrafish implicate several signalling pathways that may act to promote neurogenesis in the ENS. However, further studies are needed to tease out exactly how all the signalling pathways described above are involved both under steady state and pathological conditions.

## Conclusions and Future Directions

Enteric glia are an extremely intriguing population of cells that have a variety of roles in gastrointestinal function. Over the last couple of years, several key single cell RNA-seq studies investigating gene expression in the ENS have described the different populations of enteric neurons and their differentiation (Zeisel et al., 2018; Drokhlyansky et al., 2020; Elmentaite et al., 2021; May-Zhang et al., 2021; Morarach et al., 2021; Wright et al., 2021). Although there is also single cell RNA-seq data on enteric glia, there has as yet been no consensus linking the transcriptomic data with the current morphological classification scheme (Bon-Frauches and Boesmans, 2020). Importantly, establishing such a match will require a better understanding of the plasticity of enteric glia in the adult ENS under homeostatic conditions. The presence of the gut microbiome has been shown to be important for the attraction of enteric glia towards the lamina propria. However, whether there is movement of enteric glia within and between other niches in the intestinal wall has not been examined in detail. Furthermore, whether such mobility is accompanied with morphological, transcriptional and functional changes is not known. During development, the age-old question of how ENCC progenitors make the decision to become a neuron or a glial cell still remains largely unanswered, although we are starting to hone in on this through the various gene expression data. Lastly, enteric glia appear to act as the neural stem cell population of the gut, however in mammals, they generally remain quiescent during homeostasis. The signals that maintain low proliferation rates versus the mechanisms that induce enteric glia to proliferate and differentiate into enteric neurons remain largely unknown. How the novel findings obtained in zebrafish reconcile with mammalian models of ENS maintenance requires further investigation. Answers to these key questions will provide valuable information on the understanding of enteric glial function and their potential use in a therapeutic context.
